# Evaluating Biomarkers of Bone Health After an 8-Week Walking Program in Non-Ambulatory Stroke Survivors: A Pilot Study

**DOI:** 10.3390/jcm13216453

**Published:** 2024-10-28

**Authors:** Ramzi A. Alajam, Abdulfattah S. Alqahtani, Sanghee Moon, Caio V. M. Sarmento, Irina V. Smirnova, Marco Y. C. Pang, Wen Liu

**Affiliations:** 1 Department of Physical Therapy, College of Nursing and Health Sciences, Jazan University, Jazan 45142, Saudi Arabia; 2 Department of Rehabilitation Health Sciences, College of Applied Medical Sciences, King Saud University, Riyadh 11451, Saudi Arabia; abalqahtani@ksu.edu.sa; 3 Department of Kinesiology, University of New Hampshire, Durham, NH 03824, USA; sanghee.moon@unh.edu; 4 Department of Physical Therapy, California State University, Fresno, CA 93740, USA; caio@csufresno.edu; 5 Department of Physical Therapy, Rehabilitation Science, and Athletic Training; University of Kansas Medical Center, KS 66126, USA; ismirnova@kumc.edu; 6 Department of Rehabilitation Sciences, The Hong Kong Polytechnic University, Hong Kong 999077, China; marco.pang@polyu.edu.hk

**Keywords:** bone health, stroke, non-ambulatory, walking, bone formation, bone absorption

## Abstract

**Background/Objectives:** Stroke survivors have a significantly increased likelihood of developing osteoporosis, a condition characterized by weak and brittle bones as well as an elevated risk of bone fractures. However, previous studies on exercise intervention have mostly been on stroke survivors who are able to walk. The objective of this study was to examine the effect of walking exercise on bone health in non-ambulatory stroke survivors. **Methods:** This pre- and post-test study enrolled a group of chronic non-ambulatory stroke survivors. They were instructed to complete an 8-week aerobic walking exercise program, three sessions per week. Serum concentrations of osteocalcin (OC) and carboxy-terminal telopeptides of type I collagen (ICTP) were evaluated at baseline and after completing the walking exercise program. In addition, we assessed the ambulation capacity and balance control using the functional ambulation category (FAC) and Berg Balance Scale (BBS), respectively. **Results:** A total of 9 out of 10 non-ambulatory stroke survivors who were recruited completed the intervention. The serum concentration of OC significantly increased from 8.51 ± 2.28 ng/mL to 9.39 ± 2.97 ng/mL (*p* < 0.10). The serum concentration of ICTP significantly increased from 4.45 ± 2.58 ng/mL to 5.31 ± 2.92 ng/mL (*p* < 0.10). Both FAC and BBS scores significantly improved from 1.0 ± 0 to 1.33 ± 0.5 (*p* < 0.1) and from 7.22 ± 10.02 to 15.78 ± 14.81 (*p* < 0.01), respectively. **Conclusions:** The findings of this pilot study suggest that walking exercise may improve bone health by initiating a bone remodeling process in chronic non-ambulatory stroke survivors.

## 1. Introduction

Stroke accounts for 15–30% of disability worldwide, negatively affecting mobility and quality of life [1,2]. Stroke survivors frequently encounter secondary health complications such as osteoporosis, which is defined as decreased bone mineral and carries an increased the risk of fracture [3,4]. The prevalence of osteoporosis among stroke survivors is relatively high. A study reported that nearly 40% of stroke patients admitted to a rehabilitation center developed osteoporosis. Within six months of the onset of a stroke, there is a predominant decrease in bone mineral density, which is associated with vascular elasticity, motor impairments, decreased fitness levels, and functional limitations [5,6]. Reduced mobility and reduced weight bearing on the affected side cause loss of bone mineral density and raise the likelihood of bone fractures in stroke survivors [6,7]. In comparison to age-matched healthy individuals, stroke survivors showed a higher risk of developing osteoporosis in the first year after a stroke [8]. Studies have shown a decrease in bone mineral density and lean muscle mass in the affected lower leg compared to the non-affected lower limb in the first year following a stroke [9]. Furthermore, in comparison to healthy controls, stroke survivors exhibit significantly lower bone formation markers and significantly higher bone resorption markers [10].

The diminishment in mobility following stroke is a major factor adding to osteoporosis, as immobilization leads to significantly decreased bone mineral density. Stroke survivors who are unable to walk independently are at a higher risk of bone loss and may experience positive effects from engaging in aerobic and weight-bearing exercises, such as walking [11]. Within the first year after a stroke, non-ambulatory stroke survivors experienced a 4-fold greater decrease in bone mineral density compared to ambulatory stroke survivors [7]. Past studies have demonstrated that aerobic capacity and vascular health are related to bone health after stroke [5]. Walking training, especially with weight-bearing activities, has been found to increase bone mineral density in stroke survivors [9,11]. Furthermore, studies in individuals with spinal cord injury revealed that walking exercise with body weight support led to positive alterations in bone biomarkers, as shown by an increase in bone formation markers and a decrease in bone resorption markers [12,13]. Mechanical load and stress applied to the bones during weight-bearing activity can stimulate the factors responsible for bone growth, which in turn helps in maintaining bone density [14]. Walking training on a treadmill with harness support is probably the most effective strategy for engaging non-ambulatory stroke survivors in loading exercises.

To the best of our knowledge, no study has investigated the impact of walking exercise on bone health in non-ambulatory stroke survivors with functional ambulation category (FAC) scores of 2 or lower [15]. In addition, understanding the impact of walking training on bone biomarkers in stroke survivors is an emerging area of research that can highlight the potential benefit of this exercise on bone health. The aims of this pilot study were to investigate potential alterations in bone biomarkers and motor function, as assessed by FAC and Berg Balance Scale (BBS), following an 8-week walking exercise intervention in a cohort of non-ambulatory stroke survivors. We hypothesized that 8-week walking exercise would significantly increase bone formation markers and decrease bone markers in non-ambulatory stroke survivors.

## 2. Materials and Methods

### 2.1. Study Design and Participants

This pilot investigation was carried out with a single group. The intervention consisted of an 8-week walking exercise program comprising three sessions each week. At pre- and post-intervention time points, biomarkers for bone production and resorption were evaluated. We recruited a group of 10 stroke survivors who were unable to walk and met the following criteria: (1) achieved a score of 2 or less on the FAC, (2) were aged between 18 and 80, (3) had experienced a stroke within the past 5 years, (4) were capable of walking independently prior to the stroke, (5) were able to comprehend and follow instructions in English, and (6) were able to attend all of our intervention sessions. We excluded stroke survivors with pre-existing osteoporosis as well as those with current renal disease, unstable medical conditions, or musculoskeletal issues that could limit their participation in our intervention. All participants in our study provided informed consent prior to conducting any study procedures, and our study received approval from the institutional review board of the University of Kansas Medical Center (KUMC) (Ethical approve code: SUTY00140799; Date: 20 September 2017).

### 2.2. Outcome Measures

#### 2.2.1. Bone Biomarkers

During the pre- and post-intervention assessment days, the participants visited our study laboratory after fasting from food for a period of 12 h. A registered nurse collected a 30 mL blood sample from the antecubital vein in the unaffected arm between 8 and 10 a.m. The blood was collected into red-top tubes, specifically BD Vacutainer Serum Blood Collection Tubes. The blood collected was spun with a force of 1800 times the acceleration due to gravity for a duration of 10 min at room temperature. Afterward, it was separated, placed into a tube, and stored at a temperature of −80 degrees Celsius for future examination. Every blood sample was assigned a code and did not contain any identifiable subject information. The blood sample was analyzed to determine the serum concentration of osteocalcin (OC) and carboxy-terminal telopeptide of type I collagen (ICTP) using the Osteocalcin Human ELISA Kit (Thermo Fisher Scientific Inc., Waltham, MA, USA; catalog #: KAQ1381) and the Human ICTP ELISA Kit (Aviva Systems Biology, San Diego, CA, USA; catalog #: OKEH00680), respectively. The serum concentration of OC is positively associated with the rate of bone formation [16]. It has been used as an alternative measure for assessing the impact of anti-osteoporotic drugs [17,18]. When compared to other markers of bone resorption, ICTP, which is broken down by matrix-metalloproteases (MMPs), is more sensitive and accurate as a screening tool for bone resorption in individuals with lung cancer [19,20]. Additionally, it shows a stronger correlation with changes in bone mineral density in postmenopausal women [21]. We decided to use serum instead of urine to measure the amounts of OC and ICTP because serum markers have a lower day-to-day variability [16].

#### 2.2.2. Motor Functional Measures

After the blood sample was taken, a trained physiotherapist assessed the participants’ FAC and BBS scores. The FAC utilizes a classification system consisting of six distinct categories [15]. The lowest categorization is Category 1, denoting the inability to walk, while the highest classification is Category 6, indicating the ability to walk independently on different types of surfaces. Category 2 signifies the requirement for ongoing support and guidance from an individual to ensure safe mobility. The use of the FAC as a measure in stroke survivors has been found to be both reliable and valid [22]. The BBS is an assessment of an individual’s equilibrium, comprising 14 components. These measures evaluate sitting, standing, and weight-transfer behaviors, rating each activity on a 5-point scale [23]. The BBS has a maximum score of 56, where higher values indicate superior balance. The inter-rater and intra-rater reliability of the BBS among stroke patients has been determined to be high [24].

### 2.3. Aerobic Walking Exercise Protocol

**Figure 1:**
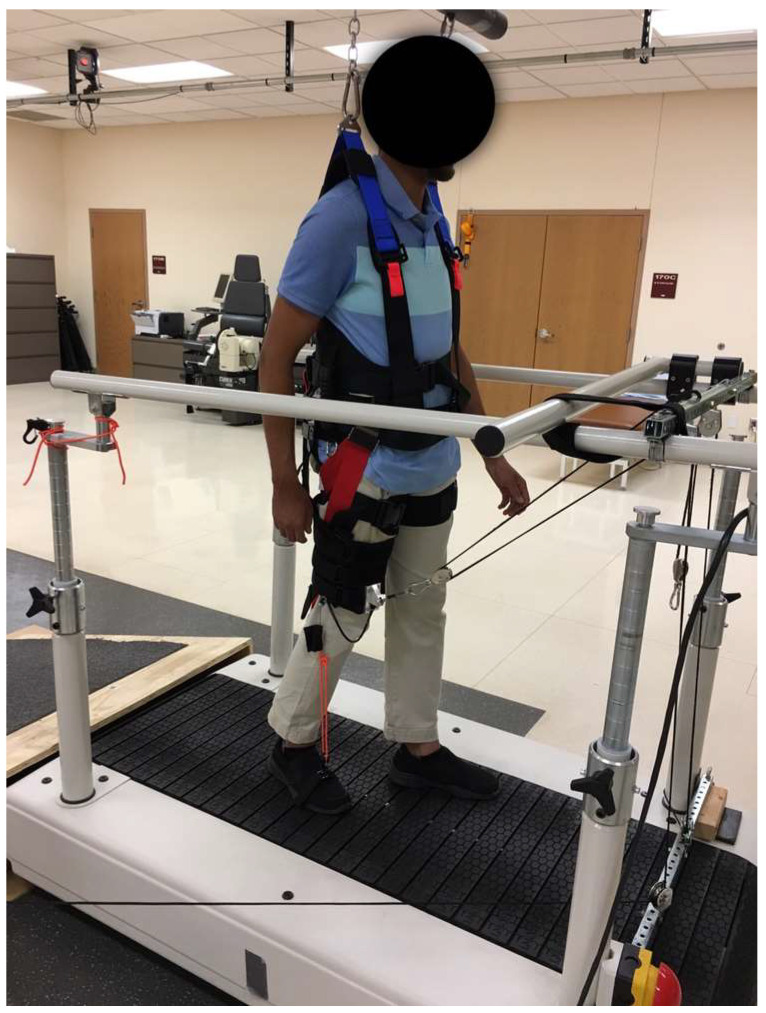
Walking-assistive device.

Throughout the course of the aerobic walking exercise, the participants engaged in walking on a treadmill for approximately 30 min, three times per week, over a period of eight weeks. A harness was secured to a ceiling lifter and fastened around the participant’s waist, thighs, and hips to provide support for a portion of their body weight and prevent them from falling. Initially, we set the body weight support (BWS) from 40–60% to ensure participant safety and comfort during training. As the participant gained confidence and improved their walking ability, we gradually reduced the amount of BWS by 5% in the following the sessions. We employed an assistive device to facilitate the participant’s progress by making the forward steps of their affected lower limbs (Figure 1). Using this apparatus, a physical therapist successfully delivered support to the affected lower limb by applying force to a cable, thereby facilitating hip and knee flexion as well as ankle dorsiflexion during the swing phase [25]. The ideal heart rate zone for achieving a low-intensity exercise was determined to be between 30% and 40% of the exercise intensity, calculated using the heart rate reserve. The heart rate reserve can be calculated using the equation [(maximum heart rate—resting heart rate) multiplied by the percentage of target exercise intensity] plus the resting heart rate [26]. The maximum heart rate was calculated using an age-predicted formula (220 minus the participant’s age) [26]. If the participant was using beta-blocker medication, we deducted 10 beats from the calculated maximum heart rate [27]. The walking exercise commenced with a two-minute warm-up period at a velocity of 0.6 miles per hour. The speed of the treadmill was gradually raised until the heart rate reached the desired heart rate zone. We concluded each walking training session with a two-minute period of cooling down. The duration of a walking exercise session was individually selected based on the participant’s tolerance level. The initial duration was set at 10–15 min and subsequently incremented by 5 min on a weekly basis until we achieved the desired duration of 30 min per session. A Polar OH1 optical heart rate sensor was positioned on the participant’s unaffected forearm to constantly record their heart rate while they engaged in walking exercise. A physical therapist positioned himself behind the participant to regulate trunk motion and provide verbal encouragement. Another physical therapist controlled the walking-assistive device by manipulating a cable to facilitate each forward step of the affected lower limb [25].

To ensure the safety of training, we checked the participant’s blood pressure prior to, at the midpoint, and at the end of each exercise session. In the event that the participant experienced fatigue, we would temporarily suspend the walking exercise for a duration of two minutes and proceed to assess the participant’s blood pressure as a precautionary step. After each exercise session was completed, we instructed the participants to assess the intensity of the activity using the rate of perceived exertion (RPE) scale. We documented the RPE, treadmill speed, percentage of body weight support, and duration of the session on a daily exercise diary after each training session.

### 2.4. Statistical Analysis

The IBM Statistical Package for the Social Sciences for Windows (SPSS) Version 25, developed by IBM Corp. in Armonk, NY, USA, was used to analyze the data. The use of descriptive statistics facilitated the reporting of means, standard deviations, and confidence intervals for all outcome measures. A paired *t*-test was conducted to compare the post-intervention measurements with the pre-intervention measurements. Due to the small sample size of the preliminary study, we set the level of significance at 0.1 [28,29].

## 3. Results

We contacted and obtained consent from ten non-ambulatory stroke survivors for our study. One of the participants withdrew from the study due to family circumstances. A total of nine participants, with an average age of 61.8 ± 13.6, consisting of five men and eight individuals with ischemic stroke, successfully completed all exercise and evaluation sessions. The data obtained from these participants were included in the final analysis of the study. Table 1 summarizes the participants’ characteristics prior to the intervention.

When comparing the findings after the intervention to those before the intervention, significant increases were observed in the serum concentration of OC (8.51 ± 2.28 ng/mL to 9.39 ± 2.97 ng/mL, *p* < 0.1) and the serum concentration ICTP (4.45 ± 2.58 ng/mL to 5.31 ± 2.92 ng/mL, *p* < 0.1) (Table 2). More precisely, the serum concentration of OC increased in five out of nine participants, whereas the serum concentration of ICTP increased in six out of nine participants. The score of FAC and BBS showed a significant improvement from 1.0 ± 0 to 1.33 ± 0.5 (*p* < 0.1) and from 7.22 ± 10.02 to 15.78 ± 14.81 (*p* < 0.01), respectively.

When comparing the data taken between the initial and final training sessions, there was a significant increase in both treadmill speed and walking duration. Treadmill speed increased from 1.0 ± 0.2 m/s to 1.6 ± 0.1 m/s, and the duration of the exercise session went from 6.8 ± 6.4 min to 29.6 ± 0.9 min. The amount of body weight support during walking exercise showed a significant reduction from 40 ± 0.0% to 10.6 ± 6.8% (*p* < 0.001). The changes made to the settings above demonstrated an improvement in the participants’ cardiorespiratory fitness.

## 4. Discussion

This pilot study aimed to investigate the alteration in bone biomarkers in non-ambulatory stroke patients following 8 weeks of walking training exercise. The findings of the study showed significant increases in OC and ICTP concentrations following the walking training. We anticipated an increase in OC concentration and a decrease in ICTP concentration. However, our findings surprised us with the apparent increase in ICTP concentration.

Studies have shown that aerobic walking activity promotes more bone production, as evidenced by a notable increase in OC serum concentration. Osteoblasts synthesize the OC, which primarily serves as an indicator of bone formation. The bone matrix integrates most of the OC and releases approximately 10% to 30% into the bloodstream [30,31]. Prior studies have documented alterations in the serum concentration of OC in non-ambulatory stroke survivors following engaging in walking training. The present study was the first to investigate changes in bone biomarkers in stroke survivors following walking training. This study found that the average OC serum concentration increased by 10.34% (0.88 ng/mL) after walking training, which was comparable to the findings of other studies conducted on individuals with spinal cord injury when accounting for a similar duration of the exercise program [12,13,32]. Prior evidence suggested that an evaluation of 10–15% of OC serum concentration from baseline measurements could be clinically meaningful, indicating a shift in osteoblast activity and overall bone formation [33,34]. This range has been associated with benefits to bone health, including improved bone mineral density and reduced risk of fractures. Among individuals with paraplegia, a 12-week body weight-assisted gait training program (consisting of three sessions per week) resulted in a significant increase in the serum concentration of OC by 0.25 ng/mL [32]. A 4-month study, which involved three sessions of supported walking training with functional electrical stimulation, significantly increased the serum concentration of OC by 1.07 ng/mL in individuals with incomplete spinal cord injury [12]. Additionally, a 6-month period of body weight-supported gait training with neuromuscular electrical stimulation increased the serum concentration of OC by 3.78 ng/mL in quadriplegic individuals [13]. The duration of the walking exercise program is a crucial element in previous studies investigating changes in OC serum concentration. According to reports, it may take 6 to 12 months or longer of weight-bearing exercise to show a significant improvement in bone mineral density [9,35]. Subsequent research might investigate the improvement of bone health in non-ambulatory stroke survivors by implementing a longer period of aerobic walking exercise intervention.

The increased ICTP serum concentration noted following the training was not expected. Over time, the serum concentration of ICTP varies in response to an exercise program. The ICTP is a peptide fragment derived from the carboxy-terminal region of type I collagen. Previous evidence describes its release from the bone matrix by MMPs, indicating bone resorption [19,36]. Studies in healthy young individuals showed an acute bout of aerobic exercise significantly increased ICTP serum concentration [37,38]. Nevertheless, the specific stage of bone remodeling that occurs in response to an exercise program greatly influences the serum concentration of ICTP. The bone remodeling process consists of five steps that occur simultaneously: activation, resorption, reversal, creation, and termination [39,40]. Initially, the activation phase begins when the body detects the presence of early remodeling signals. For instance, osteocytes detect mechanical load and stress changes in the skeleton during exercise and, upon reaching specific thresholds, trigger bone remodeling [41]. Subsequently, a phase of bone resorption occurs, lasting approximately two weeks, in which osteoclasts attach to the surface of the bone and break it down. Following that, a reversal phase, lasting approximately one month, transforms bone resorption signals into markers of bone formation. The bone formation phase, lasting approximately four months, carries out the full replacement of osteoclastic cells with osteoblastic cells and achieves mineralization prior to the termination phase. We speculate that the increased ICTP serum concentration observed in the current study indicates either a resorption or a reversal phase of the bone remodeling process at the end of the 8-week walking exercise. Our speculation is based on the following facts. Our participants were non-ambulatory, with an average 27 months after onset of stroke. Their bone quality, especially in lower extremities, would have declined significantly due to their sedentary lifestyle and lack of mechanical loading. During the 8-week walking exercise program, the averaged treadmill speed increased by 60%, walking duration within a session increased by 4.4 times, and body weight support decreased from 40% to around 10% of the body weight. Due to the slow treadmill speed, short walking duration, and significant unloading assistance, the early walking exercise sessions may not generate a large enough mechanical load to trigger the bone remodeling process. We believe that the late sessions would generate sufficient loading to initiate the bone remodeling process, as shown in the increased ICTP serum concentration. Since the OC concentration significantly increased at the end of the intervention, it is more likely that the bone remodeling process reached the reversal phase. Further studies are necessary to directly examine the changes in bone density over a long-term duration of training, along with follow-up assessments.

The relationship between better walking capacity and improved bone health is closely connected. With improved mobility, stroke survivors can engage even more in weight-bearing activities, which are necessary for promoting bone growth. In our study, a number of participants demonstrated improvements in their walking capacity following training. This was evidenced by a 0.33 increase in the mean FAC score at the end of training. Previous studies have reported an improvement in walking ability among non-ambulatory stroke survivors following treadmill walking training [42]. Additionally, a study by Cho et al. [43] found that training modalities such as robotic-assisted treadmill walking training further improve walking capacity and balance, which are essential for maintaining bone health. They reported a mean change of 0.4 in the FAC score, which was similar to the observed mean change in the current study (0.33).

Our participants’ enhanced ability to control balance may be associated with their improved walking ability. Stroke survivors who have substantial impairments experience impaired balance control, which hinders their ability to engage in daily physical activities and locomotion due to fear of falling [44]. A past study observed positive correlations between walking ability and balance control among chronic stroke survivors [45]. Evidence has proven that walking training is a highly effective method for improving balance in stroke survivors [40]. Walking exercise, which focuses on the unaffected lower extremity, can improve balance in chronic stroke survivors who can walk independently [44]. In addition, dynamic standing therapy and gait robotic gait training can improve balance control in stroke survivors who are unable to walk, as assessed by the BBS [43,46]. During training, our participants were required to fully bear weight on their unaffected lower limbs while simultaneously maintaining balanced control of their trunk. Consequently, their ability to maintain balance was enhanced over time. Improved balance control following walking training can reduce the risk of falling, indirectly benefiting bone health by preventing fractures.

We noticed various limitations in this study. First, we should approach the current study’s findings with caution due to its limited sample size and lack of a control group. Second, the study analyzed a single bone formation marker, osteocalcin (OC), and a single bone resorption marker, C-terminal telopeptide of type I collagen (ICTP). Incorporating additional markers of bone formation and resorption can provide a more comprehensive understanding of how walking exercise affects bone in non-ambulatory stroke survivors. Third, we did not account for the influence of drugs, such as vitamin D supplements, that could potentially impact bone quality [4,47]. Furthermore, we did not account for the potential impact of other drugs, such as some diabetes medications, that may contribute to bone density loss [48]. The consumption of foods rich in calcium or vitamin D was not controlled in the study participants. Lastly, we did not account for additional variables, known as covariates, that could potentially influence the outcomes of this study. These covariates include diabetes [49], the amount of time after the stroke onset [50], sex hormones [34], level of physical activity [6], and the season in which blood samples were collected [16].

## 5. Conclusions

The findings of the study suggest that 8-week walking exercise program successfully enhances bone health in chronic non-ambulatory stroke survivors. At the end of the walking exercise, there was a notable increase in both markers of bone formation and resorption, indicating the initial stage of bone remodeling. Subsequent investigations should be conducted with a more extensive sample size, inclusion of a control group, and extended follow-up assessments in order to validate the results obtained from this preliminary study.

## Figures and Tables

**Table 1:** Participants characteristics.

Age (Years), Mean ± SD (Range)	61.8 ± 13.6 (40–78)
Gender, n (%)	
Male	5 (55)
Female	4 (45)
Type of stroke, n (%)	
Ischemic	8 (88)
Hemorrhagic	1 (11)
Hemiparetic side, n (%)	
Right	8 (88)
Left	1 (11)
Time since stroke (months), mean ± SD	27 ± 14.3
Race, n (%)	
White	5 (45)
African-American	3 (33)
Other	1 (11)
Body mass index (kg/m^2^), mean ± SD	28.6 ± 8.2
Hypertension, n (%)	
Yes	8 (88)
No	1 (11)
Diabetes mellitus, n (%)	
Yes	3 (33)
No	6 (66)
Vitamin D supplements	
Yes	5 (55)
No	4 (45)
Anti-depression drugs	
Yes	4 (45)
No	5 (55)

**Table 2:** Mean and standard deviation of bone biomarkers, walking capacity, and balance.

					90% CI of the Difference		
	Pre-Intervention	Post-Intervention	Mean Differences	Effect Size	Lower	Upper	*p*-Value
OC (ng/mL)	8.51 ± 2.28	9.39 ± 2.97	0.88	0.48	−0.27	2.03	0.09 *
ICTP (ng/mL)	4.45 ± 2.58	5.31 ± 2.92	0.87	0.90	0.27	1.47	0.02 *
FAC	1 ± 0	1.33 ± 0.5	0.33	0.66	0.02	0.64	0.08 **
BBS	7.22 ± 10.02	15.78 ± 14.81	8.56	1.57	5.17	11.94	<0.01 **

Values are presented mean ± standard deviation; OC: osteocalcin; ICTP: carboxy-terminal telopeptide of type I collagen; FAC: functional ambulation category, BBS: Berg Balance Scale; * statistically significant, *p* < 0.01 (one-tailed *t*-test); ** statistically significant, *p* < 0.01 (two-tailed *t*-test).

## Data Availability

The raw data supporting the conclusions of this article will be made available by the authors on request.
